# Political challenges to prioritizing gender in global health organisations

**DOI:** 10.7189/jogh.10.010702

**Published:** 2020-06

**Authors:** Yusra Ribhi Shawar, Jeremy Shiffman

**Affiliations:** 1 Johns Hopkins University, Bloomberg School of Public Health, Department of International Health, Baltimore, Maryland, USA; 2 Johns Hopkins University, Paul H. Nitze School of Advanced International Studies, Washington, D.C., USA

## Abstract

**Background:**

Many global health organisations have adopted formal strategies to integrate gender in their programming. In practice, few prioritise the issue. Institutions with considerable global power therefore largely overlook fundamental drivers of adverse health outcomes: gender inequality and harmful gender norms. We analyse the factors shaping attention to gender in organisations involved in global health governance.

**Methods:**

Drawing on scholarship from the fields of organisational behavior and management, sociology, international relations and the policy process, we undertook a thematic analysis of peer-reviewed scholarship and organisational documents. We also conducted 20 semi-structured interviews over Skype with individuals working at the cross-section of gender and health.

**Results:**

In seeking to reform the policies and practices of global health organisations, gender proponents confront patriarchal organisational cultures, hostile political environments and an issue that is difficult to address as it requires upsetting existing power structures. Proponents also face three linked challenges internal to their own networks. First, there is little cohesion among champions themselves, as they are fragmented into multiple networks. Second, proponents differ on the nature of the problem and solutions, including whether reducing gender inequality or addressing harmful gender norms is the primary goal, the role of men in gender initiatives, which health issues to prioritise, and even the value of proponent cohesion. Third, there are disagreements among proponents on how to convey the problem. Some advance an instrumental case, while others believe that it should be portrayed as a human rights issue and using any other argument undermines that fundamental justification.

**Conclusions:**

Prospects for building more gender-responsive global health organisations will depend in part on the ability of proponents to address these disagreements and develop strategies for negotiating difficult organisational cultures and political environments.

Many organisations involved in global health governance have adopted formal gender strategies that seek to mainstream gender in activities and programs, address inequalities, ensure gender-responsive health delivery, and strengthen responses for women, men, girls, boys and non-binary people. In practice, however, few prioritise the issue [1-5]. Institutions with considerable power to shape global responses to health problems therefore largely overlook gender inequality as a fundamental driver of health inequities and adverse health outcomes.

The evidence on neglect is extensive. Funding for the issue, while growing, is insufficient [6]. Men far outnumber women in high-level leadership positions in these organisations [1,3,7]. Rather than hire gender experts as core staff, organisations sometimes rely extensively on outside consultants or allocate gender specialist responsibilities to those already holding full-time positions, often with no extra compensation or support [8]. Little of the health policy, programming and research that these organisations design or support incorporate gender in a meaningful way [1,3,9]. When they do mention gender, strategy documents often are process-oriented rather than focused on health outcomes and/or inappropriately equate gender and health with women's health [1,10]. Gender-tracking methodologies rarely monitor program implementation [8]. And most recently, institutional strategies to address the Ebola and Zika epidemics lack gender indicators and consideration of the gendered impacts of these diseases [11,12].

We analyse the factors shaping attention to gender in organisations involved in global health governance (hereafter referred to as global health organisations). These include multi-and bi-lateral organizations, international non-governmental organizations, large philanthropic and other funding agencies. Gender refers to the “culturally defined roles, responsibilities, attributes, and entitlements associated with being (or being seen as) a woman or man in a given setting, along with the power relations between and among women and men”[13]. Beyond promoting gender equality in its discourse and practice, attention to gender implies that an organisation systematically accounts for gender-related risks in the design, execution, and evaluation of its policies and programs, challenges restrictive gender norms – “the often unspoken rules that govern the attributes and behaviours that are valued and considered acceptable for men, women, and gender minorities” [13] – that result in negative health outcomes, and allocates sufficient funding and human resources to support such efforts. A clear understanding of these factors is essential for ensuring priority for the issue during the Sustainable Development Goal (SDG) era.

Drawing on scholarship from the fields of organisational behavior and management, sociology, international relations and the policy process, we identified four categories of factors as a useful way to examine attention to gender. These are: (1) organisational culture – the set of shared assumptions and values, as reflected, for example in an organisation’s rules, accountability mechanisms, and incentive structures, that define appropriate behavior for individuals in organisations; (2) political contexts – the environments in which these organisations operate; (3) issue characteristics – features of the problem; and (4) proponent power – the strength of concerned individuals.

Factors connected to organisational culture help explain why organisations prioritise some issues and neglect others. Organisational imprinting theory suggests that the context at the time of an institution’s founding “imprints” an enduring culture, with lasting effects on priorities, strategies and operating practices [14]. Other forces, such as the interests and ideologies of powerful actors within these institutions, establish influential behavioral guidelines beyond founding conditions, solidifying the imprinting effect and shaping organisational choices even as external conditions change [15]. Professional culture also shapes organisational priorities [16,17]. Professionals hold distinctive worldviews and normative commitments that vary by discipline. For instance, economists are trained to analyze cost-effectiveness and to value efficiency. When dominant in a particular organisation, professionals of a particular discipline may shape priorities along the lines of their training and values [17,18]. These various forces shape employee behavior by establishing an organisation’s practices and processes, as well as its incentive structure – criteria for rewards and sanctions.

The political contexts in which institutions operate also influence organisational priorities. States create international organisations such as the World Bank, and expect them to act in accordance with their national interests [19]. The preferences of powerful states, such as the United States, may be particularly influential. These states may compel international organisations to prioritize those conditions they most fear (for instance, communicable diseases) to the neglect of other problems.

Sociologists and political scientists point to another external political force that may shape organisational agendas: the spread of ideas and norms – standards of appropriate behaviour for actors with a given identity [20] – through the international system. A norm follows a life cycle with three phases [21]: 1) norm emergence, when entrepreneurs identify and frame a problem, seeking to convince states to change policy; 2) norm cascade, when the norm reaches a tipping point as a critical mass of states embrace the norm; and 3) norm internalization, when the norm is codified, universally adhered to and taken for granted. Several factors may increase the likelihood that a norm reaches a tipping point: (1) the presence of capable norm entrepreneurs; (2) the availability of organisational platforms to advance their case; (3) the grafting of the new norm onto existing, related, widely accepted norms; (4) a focus on issues with widespread resonance such as legal equality of opportunity or bodily integrity; and (5) an external shock, such as an economic depression, that pushes states to look for new policy ideas [21].

Issue characteristics also shape organisational priorities. Organisations are more likely to prioritize issues that are soluble and have simple chains of causation. Also, they are also more likely to provide attention to issues that are perceived to cause extensive harm, that are politically uncontroversial, and that affect a sympathetic population group [22-24].

An additional consideration is the capacity of proponents concerned with the issue to pursue collective action [24-28]. Networks of proponents commonly face several challenges in this regard [29-34]. Among these are cohesion – generating coalescence among the network of individuals and organisations that are centrally involved with the issue; problem definition – forging consensus on what the problem is and how it should be addressed; positioning – portraying the issue in ways that inspire external audiences to act; and governance – building effective institutions to achieve collective goals. Key considerations for building effective network governance include: cultivating internal legitimacy through the development of trust and effective communication mechanisms among members [35], building external legitimacy by mobilizing new members and resources and promoting the network’s activities to outside stakeholders [36], and maintaining a balance that enables the network to be stable at its core and flexible at its periphery [37].

## METHODS

Our analysis is based on a triangulation of peer-reviewed research, organisation reports, and grey literature, as well as semi-structured interviews with individuals in global health organisations working at the cross-section of health and gender, and with critics and observers of their efforts (see **Table 1** for organisational affiliations of respondents). The study protocol underwent ethics review and was approved by the Institutional Review Board of American University (Washington, DC, USA).

**Table 1:** Organisational affiliation of key informants

Organisational affiliation of key informants
American University
Asian Development Bank
Bill and Melinda Gates Foundation
Breakthrough US
Center for Global Development
Center for Health and Gender Equity (CHANGE)
EngenderHealth
Gender@Work
Global Forum for Health Research
Harvard University
Interagency Gender Working Group
International Center for Research on Women (ICRW)
International Development and Relief Foundation
Institute of Development Studies
Inter-American Development Bank
Jhpiego
John D. and Catherine T. MacArthur Foundation
Johns Hopkins University
MenCare
MenEngage
PATH
PEPFAR
Population Council
Promundo
Save the Children
Sree Chitra Tirunal Institute for Medical Sciences and Technology
Queen Mary University of London
The United Nations Population Fund (UNFPA)
United Nations Children’s Fund (UNICEF)
United Nations Secretary General’s Men’s Leaders Network
United Nations Women (UN Women), formerly United Nations Division for the Advancement of Women (UNDAW)
United States Agency for International Development (USAID)
University College London
University of Bristol
University of Cape Town
University of the Western Cape
University of Sydney
Wellcome Trust
William and Flora Hewlett Foundation
Women Win
World Bank
World Health Organization (WHO)

### Interviews

We conducted 20 semi-structured interviews. Employing a purposive rather than representative sampling strategy, we identified key informants through consultation of published and grey literature on gender in global health, and by asking interviewees whom they considered most centrally involved in shaping consideration of gender within global health organisations. We conducted interviews over the phone between November 2016 and April 2017. These lasted approximately one hour on average. A sample questionnaire is included in the appendix. We continued to interview key informants until we reached theoretical saturation – the point at which we obtained no new critical information from additional data. All interviews were recorded and transcribed with consent from participants. The detailed notes taken during the interviews, audio recordings, and audio transcriptions were de-identified and secured to ensure respondent confidentiality. We assigned a key informant number to each individual, which is referenced in the presentation of the findings (see **Table 2**).

**Table 2:** Key informant number/ organisational type/HIC/LMIC

Key Informant Number	Organisational Type	HIC/LMIC
1	Academic	HIC
2	Academic	HIC
3	UN Agency	HIC
4	UN Agency	HIC
5	NGO	HIC
6	Academic	HIC
7	NGO	HIC
8	Academic/UN Agency	LMIC
9	Academic	HIC
10	NGO	HIC
11	Foundation	HIC
12	NGO	HIC
13	NGO	HIC
14	Academic	LMIC
15	Academic	LMIC
16	UN Agency/Foundation	HIC
17	UN Agency	HIC
18	UN Agency	LMIC
19	UN Agency	HIC
20	NGO	HIC

HIC – high-income country, LMIC –low- and middle-income country, NGO – non-govermental organization, UN – United Nations

### Literature review

We also examined peer-reviewed research, organisation reports, and grey literature (n = 192). We searched Google Scholar, ProQuest, JSTOR databases and global health organisational websites. The search was restricted to literature in English, between the years 1980 and 2019. The search terms used were: “gender”, “gender equality”, “gender justice”, “gender norms”, “women empowerment”, “women/woman” and/or “men/man”, in combination with “global health”, “health”, “strategy”, and/or “policy”. We also included the names of major global health organisations in our search, including international organisations, UN agencies, philanthropies, government institutions, and non-governmental organisations that contribute significant funds or are involved in large-scale implementation of programs in the area of health.

### Qualitative analysis

We undertook a thematic analysis of the collected information, using an iterative process in developing the codes [38]. We originally coded data by two broad categories derived from policy frameworks that examine the determinants of political priority for global health issues: proponent power (the strength of involved actors and the way in which they understand the problem and portray it to decision-makers) and political contexts (the normative and policy environment in which actors operate) [24,34]. The coding evolved to also include an examination of the organizational culture (the embedded rules, assumptions, and values in global health organisations) and issue characteristics (the nature of the problem). Rather than take a stance on normative debates, we sought to analyze, for example, how organisational strategies and policies emerged and developed; the influence of external political developments, prominent reports and emergent data; and the major points of disagreement and consensus among those interested in advancing the gender agenda, as well as the perspectives of those critical of it. We triangulated the data from the literature review and interviews in order to minimize bias and validate the accuracy of the analysis. We also received feedback on the paper from seven individuals, four of whom participated in key informant interviews.

## RESULTS

In seeking to reform the policies and practices of global health organisations, advocates concerned with gender and health face several external barriers. These pertain to organisational culture, political environments and issue characteristics. They also face challenges internal to their own networks, pertaining to cohesion, problem definition and positioning. We refer to these advocates broadly as “gender proponents” rather than “gender equality proponents” or any other more specific name. We do so because, while gender equality is a focal concern for many actors, it does not encompass the entirety of the gender agenda (for instance, some actors are concerned with the impact of toxic masculinities on the health of boys and men).

### External challenges

#### Organisational culture

Patriarchal and sexist organisational cultures hinder the efforts of gender proponents. Practices, including bias favouring males, are often long-standing, entrenched and taken for granted [39,40].

Few gender specialists hold positions of power in these organisations (Interview (I)1, I6, I7, I8, I14, I17), and many of their colleagues see little value in applying a gender lens (I1, I5, I7, I8, I15, I17, I20). Gender specialists describe feeling intimidated and marginalized (I1, I4, I6, I7, I8, I11, I14, I17, I19, I20):

“We are not part of the boys’ club….You are yelled at and talked to disrespectfully in ways I don’t think some would do with male peers” *(I14).*

“Those genuinely working on gender equality are pushed in a corner...the geeks who drill a hole in your head wanting to have the document with a gender lens” (*I17).*

“I had one very senior person – a woman – say, ‘There are no gender issues in my life. I don’t know why you’re still talking about gender equality. All of that has been resolved’” *(I8).*

Gender specialists also describe feeling overextended (I1, I7, I8, I16) [41]. Some of this may stem from workplace policies that do little to promote gender equality, support women's careers, encourage work-life balance, and address instances of sexual harassment. It is also due to organisations assigning individuals already holding full time jobs (such as human rights focal points) to be gender specialists. The jobs of gender specialists are all the more difficult because many are often hired with little support or resources. Some go so far as to describe their hire to be for cosmetic purposes – to make their organisations appear to be taking gender seriously (I7, I11) [42].

Leadership and career prospects for gender specialists (nearly all of whom are female) and other women are poor, despite some improvement over time [1,7]. Although women constitute over three-quarters of the health workforce, many organisations have never had a top executive who is female (including the World Bank, the Global Fund to Fight AIDS, Tuberculosis and Malaria, GAVI and UNAIDS) [43]. Only one in ten awards in the fields of medicine and public health are given to women [44]. Also, women who identify as feminists report discrimination, making career advancement difficult (I1, I8, I11, I16) [45]. One comments:

“If you’re a woman who says you’re a feminist, it can actually go against you because it suggests that you’re only interested in women’s issues….It’s one thing for a man to say he’s a feminist and another for a woman” *(I1).*

Disciplinary norms pose an additional barrier to prioritizing gender (I1, I2, I4, I5, I8, I16, I18, I19, I20). With some exception, few of the physicians, public health professionals and economists who comprise the majority of the leaders and managers in these organisations have been socialized to value gender analysis. Many emphasize technical solutions and quick fixes rather than consideration of the root causes of inequality and ill health that gender analysis emphasizes [10]. Much medical, nursing, dental, pharmacy, and public health training rarely considers sex or gender beyond the physiology of reproduction and often fails to take the cultural, social, and economic assumptions and impacts on an individual’s health into account (I8) [46]. One respondent conveys her perception of the technical worldviews of many professionals in these global health organisations:

“People are bleeding; we need to be able to close those wounds. People are dying; we need to be able to fix this problem. People are starving; we need to be able to get food to those people. [All this] without recognizing those underlying factors that are creating poor health outcomes to begin with” *(I5).*

#### Political environments

In addition to organisational cultures, features of the global political environments that surround these organisations have hindered their prioritization of gender. While some governments champion the issue, conservative governments resist the agenda – many viewing gender equality to be a Western imposition and/or a threat to their cultures – prompting global health organisations to act cautiously for fear of offending key constituents (I8, I13) [47]. For instance, in the early 2000s a number of Islamic states and the United States under the George W. Bush administration blocked inclusion of reproductive health in the Millennium Development Goals (MDGs) [48]. Most recently, proponents fear that the international wave of populist politics will inhibit global health organisations (I1, I13, I14, I15, I16, I19), one commenting that (I15), “politics is throwing up more and more rightwing misogynist leadership.”

An additional impediment are historical initiatives that have led to a conflation of gender with a concern for women (I19) [49]. In the 1970s, the women’s movement called upon international development agencies and governments to integrate women into the development process, leading to the United Nations Decade for Women and subsequent World Conferences on Women. Due to the slow pace of progress in improving women’s status and well-being, the idea of gender mainstreaming emerged in the mid-1980s [50]. While there has since been a gradual shift in both academic and policy circles from a conceptual focus on women (as represented by the Women in Development movement) to a focus on gender (as represented by the Gender in Development movement), confusion lingers about the practical differences. Among the consequences of this confusion have been insufficient consideration of gender norms and power relations, and minimal uptake of considering masculinities in international development practices and policies [10,50-52].

Also, the MDGs reinforced a narrow understanding of gender by largely ignoring complex power relations, promoting a limited set of indicators that encouraged a “quantified numbers game” (I1), and pursuing a categorical approach that targeted women separately from men (I7, I17) [53]. Among the consequences were efforts to address maternal mortality that failed to consider root causes, including the low social value placed on women’s lives.

Many gender proponents see this conflation of gender and concern for women as undercutting the application of what they call a true gender lens, and bolstering instead the examination of sex differences from a biological perspective (I1, I3, I13, I16, I17, I19). Respondents express their frustrations:

“I think a lot of institutions have gender as code for ‘women’” (*I3).*

“People feel that if they are doing women’s health, they are doing work on gender” *(I19).*

Moreover, global health organisations often limit their consideration of gender to reproductive and sexual health, violence and infectious diseases (mainly HIV/AIDS, tuberculosis, and malaria), to the neglect of non-communicable diseases, epidemics, injuries and other conditions shaped heavily by gender norms and power relations (I1, I8, I11) [1,12,54]. There are, however, exceptions (eg, the WHO published a series of policy briefs in the 2000s applying a gender lens to several different health issues, including tobacco control (2007) and water and sanitation (2006)).

#### Issue characteristics

The nature of the issue itself also hinders prioritization of gender considerations in global health organisation programs and practices. Critics claim ‘gender’ is ambiguous, complex, and academic (I3, I5, I7, I12, I19, I20) [41]. One respondent notes:

“The word gender is off-putting and different people interpret it to mean different things. As a term it is also hard to operationalize in practice” *(I3).*

Another respondent notes an implication of this alleged opacity, contrasting the issue with more easily grasped problems such as violence against women (I19): “It’s really hard to grow a movement around gender.” A related difficulty is gender’s cross-sectoral nature. Like nutrition, health systems strengthening and environmental sustainability, addressing gender requires engagement with multiple technical areas. Gender is the responsibility of many actors, making it susceptible to being the responsibility of no one [55].

Most challenging, unlike many global health problems such as vaccine delivery, addressing gender inequalities requires fundamentally upsetting existing power structures (I5, I7, I11, I12). As respondents note:

“We’re talking about changing the sexual division of labor. We’re talking about changing gender power relations. We’re talking about very threatening ideas. And there are consequences for people” *(I7).*

“These are deeply entrenched ways of being…people are hesitant to step into that complex muddy grey space and work to see the transformational change that is needed” *(I5).*

Often, the politically powerful individuals able to influence the deep structures that keep gender inequality in place have little incentive to do so, while the populations most adversely affected are politically marginalized (I12). Recognizing this challenge, many global health organisations adopt technocratic or integrationist approaches to gender – adding it on to organisational activities – rather than a transformative approach that aims fundamentally to alter institutional practices and norms (I5) [10,41].

### Internal challenges

While many impediments to addressing gender stand external to proponents, some concern the proponents themselves. These linked internal challenges pertain to cohesion, problem definition and positioning.

#### Cohesion

Tight communities work actively to address some concerns in global health, including tobacco control, tuberculosis and newborn survival [56-58]. By contrast, gender proponents do not constitute a cohesive community with clear boundaries. They are dispersed, situated in at least three overlapping, informal networks – addressing gender and health, men and masculinities and health, and women’s health. Moreover, the extent to which individuals in these networks consider gender to be core to their professional identity varies: for some it is central (ie, gender specialists); for others it is important but not primary (ie, many maternal health specialists). The absence of clear community boundaries offers the advantage of bringing in diverse perspectives; it also makes collective action challenging.

Of the three networks mentioned above, the one most centrally concerned with gender broadly understood is a group of individuals working under the guise of gender and health. Many are academics who gather at forums on women’s health (I8). Most gender specialists within global health organisations are also part of this network. These individuals are concerned with the ways in which societies assign roles, responsibilities and expectations to men and women, and the influence of these social constructions and of gender relations on health outcomes. Many identify with a larger concern surrounding gender and development, whose roots lie in part with the women’s movement (I4, I8, I19). The focus of these individuals is broader than the health of either sex or a particular issue such as HIV/AIDS.

A second network, emerging in the late 1990s, is comprised of individuals concerned with masculinities and health. The masculinities and health movement is two-pronged in nature. One group has sought to raise the profile of men’s and boys’ contributions to women’s health, advancing the critical role that men and boys have in transforming harmful gender norms and unequal power dynamics [59]. The other group is centrally focused on the well-being of men and boys with an emphasis on addressing the negative effects of gender norms pertaining to masculinity [60-62]. Members of the former group are deeply committed to feminist goals and view their efforts as building on the women’s movement. Members of the latter group, while not opposed to these goals, focus on problems such as ‘men in crisis,’ ‘troubled masculinities,’ and ‘men at risk’, and the fact that younger, low-income males are marginalized and live with insecurity [63]. Core individuals are based in Australia, Ireland, and the United States (ie, Australian Men’s Health Forum, Men’s Health Forum in Ireland, Men’s Health Network (USA), Men’s Health Education Council/Prostate Conditions Education Council (USA)). However, the group is expanding globally, as evidenced by the 2014 launch of the Global Action on Men’s Health, an initiative aimed at addressing the poor health of men across the world [64].

The largest of the three networks is comprised of academics, advocates and practitioners addressing the health and well-being of women and girls. Some individuals are concerned with this area broadly, but most focus on one or more specific issues, including maternal health, sexual and reproductive health, violence against women, and HIV/AIDS. Most claim a concern with gender, but unlike many members of the other two networks, few identify gender equality as their central focus. Several forums have brought these women’s health proponents together (as well as facilitated coalescence of the other two networks). These include the 1994 International Conference on Population and Development (ICPD) in Cairo, the 1995 Fourth World Conference on Women in Beijing, the Association for Women's Rights in Development (AWID) international forums, and more recently, a series of Women Deliver conferences and a 2012 Commission on Women and Health [65,66].

#### Problem definition

Differences among gender proponents concerning the nature of the problem and solutions contribute to this lack of cohesion (I2, I3, I4, I6, I7, I8, I10, I11, I12, I13, I14, I15, I17, I18). One gender proponent describes the situation:

“You might imagine that they could be unified under an all-encompassing theory of gender but that’s not what really happens” *(I9).*

One point of disagreement is whether reducing gender inequality or addressing restrictive gender norms should be the primary goal (I4, I6, I9). Proponents advancing the former – individuals predominately involved in women’s movements – see the need to complete the unfinished agenda of achieving gender equality. Proponents supporting the latter – newer to the field of gender and health – argue that restrictive gender norms adversely affect both women and men, and see a need to focus on gender to improve well-being for all populations. Still others don’t see addressing gender inequality or restrictive gender norms as “mutually exclusive”, since “gender norms underpin gender inequality”, which “can work against men although historically it is women who have been disadvantaged” (I3).

A related concern are the subjects of male health and masculinities, and the role of men in gender initiatives. A number of proponents are uneasy about those advancing a concern for masculinities in global health and development, worried that they may ‘male-hijack’ the gender agenda, encroach on limited funds, and undermine work focused on women [67]. Proponents differ also on how and the extent to which men should be engaged in advancing gender equality (I5, I8, I10, I13, I15), some arguing that doing so undercuts the empowerment of women and girls. Others argue that involving men in global and national initiatives, and engagement with men and boys in transforming harmful gender norms and power dynamics, is critical to the health and well-being not just of men and boys but also of women and girls.

Some proponents argue that the very concept of “gender norms” is ambiguous, that norms are only one determinant of health outcomes, and that an excessive focus on these will lead policy-makers to believe that there are simple solutions to gender problems in health (I6, I11, I12) [68]. As one respondent notes:

“Norms are, at most, one among a number of determinants of action… My guess is that the policy language of norms has mainly come from global North contexts. In global South contexts, issues like violence, poverty and power, rather than norms, leap to the eye when thinking about health issues such as AIDS, malnutrition, refugees, poor housing and sanitation, and pollution” *(I6).*

Yet another tension is over which health issues to emphasize (I4). Gender proponents historically have focused on violence against women and issues connected to reproduction, including sexual and reproductive health and rights, women’s vulnerability to HIV infection and maternal survival. Some proponents are critical of this extensive focus on reproductive issues, arguing that it crowds out attention to other pressing concerns such as non-communicable diseases (I8). A related tension is the extent to which to engage politically controversial subjects such as abortion access and LGBTQ rights. While most gender proponents agree that these concerns are critical for achieving gender equality, for tactical reasons some argue setting these aside temporarily to ensure progress on other aspects of the agenda. Others contend that it is a disservice to the end goal of gender equality when certain issues are separated out and “thrown under the bus” (I13).

Proponents even diverge on the value of cohesion. Some see disagreements among gender proponents as a problem [69]. One respondent worries about losing public sympathy and scarce resources as proponents “fuss over the size of the pie and who gets a slice” (I9). Others question the value of having unity of voice, identifying diversity as strengths of gender networks (I7, I12, I14, I15). As one notes:

“I don’t think there’s necessarily a relationship between cohesion and influencing others. I think that the beauty of feminist movements is that they are spaces of freedom and respect for multiple identities and ideas and framings…” *(I7).*

#### Positioning

In addition to concerns about problem definition, gender proponents also differ on positioning – how they should convey the issue to policy-makers and other external audiences whose resources and support are needed to advance the gender and health agenda. These differences affect network cohesion and agenda prospects.

Most proponents are motivated by the goals of advancing human rights, dignity and capabilities. They differ on whether to use instrumentalist framings to achieve these ends. Some respondents argue that economic and other arguments are essential since many policy-makers are unresponsive to rights claims (I10, I11, I16, I17) [70]. As one respondent puts it:

“For decades, the reproductive health community – I’m one of them – went around using this moral argument: ‘How could they [women] die when they are giving life to somebody else?’ We got nowhere. Do you know what got us somewhere? The economics of it. When we started costing – what does it mean for a woman to die in childbirth?” *(I17).*

Other proponents are uneasy about the use of instrumentalist framings that do not align with their core beliefs (I1, I7, I11, I13). Moreover, they are concerned that an instrumentalist treatment of gender in global health organisations will not likely lead to transformative outcomes (I7). One respondent comments:

“[During] my entire feminist organizing life people keep saying what’s the business case? It is the most irritating response…We don’t have to make a business case. There is no business case for going to church. You do it. You do it because you believe in it, because you signed up” *(I7).*

An additional tension concerns the use of a ‘women’s empowerment’ framing (I4, I6, I9, I13, I15). Some proponents are uneasy about its use. They worry that such a framing undermines a concern for gender equality broadly and reinforces a propensity for global health organisations to equate gender to women and to fixate on sex differences. Others, however, argue for use of this framing because it is easier to understand than ‘gender’, believe it to be effective, and contend that a focus on women will achieve similar outcomes (I4).

These positioning debates shape how proponents view the work of gender specialists inside global health organisations. Some argue that the promotion by gender specialists of business and other instrumentalist arguments renders them “complicit in neo-liberal restructuring” [71]. Others argue that such a view overlooks the real constraints placed on these cadres of professionals who face strong resistance to promoting gender equality [41]. As one respondent notes:

“My impression is that UN, INGO and government discussions of gender issues have been significantly shaped by femocrats’ and activists’ judgments of what they can get male ministers, parliamentarians, ambassadors and senior bureaucrats to understand about gender – and that is, frankly, very little. Categorical statistics of inequality are therefore prominent. I do this myself; if I want to catch a reader’s attention, I point out that 96% of the CEOs of the top 500 transnational corporations are men” *(I6).*

## DISCUSSION

Few global health organisations consider gender systematically in their policy-making and programming. They are impeded by their organisational cultures, political environments and characteristics of the issue. Among the barriers are entrenched, patriarchal practices; lack of training of professionals in these organisations to value gender analysis; conservative governments that view gender equality as a Western imposition; fear by government and global health officials that addressing gender will upset existing power relations; a conflation of gender with a concern for women and girls; and the complexity of the concept. Other barriers concern the proponents themselves: the absence of a cohesive community; differences among them on the nature of the problem and solutions, including which health issues to prioritize; and divergence on how to position the issue, particularly surrounding the use of instrumentalist, economics-oriented arguments.

Despite these challenges, there are reasons for optimism. A growing number of global health organisations have adopted gender strategies, including UNAIDS [72], UNICEF [73], the WHO [74], the World Bank [75] and the Global Fund to Fight AIDS, Tuberculosis and Malaria [76]. Global Health 50/50 launched in 2018 to address gender inequalities in global health institutions. SDG 5 is dedicated to gender equality and women’s empowerment. Right-wing politics have inspired counter-mobilization, including women’s marches in 61 countries after US President Trump’s inauguration [77]. The quality, availability, and use of sex disaggregated data with a gender analysis are increasing [78,79]. In addition, despite differences on problem definition, solutions and positioning, proponents are unified by a shared commitment to ensuring that global health organisations take gender seriously.

Prospects for building more gender-responsive global health organisations will depend on change in the external political environment, organisational cultures and the strategies proponents adopt to pursue collective action and shape perceptions of the issue.

### Changing the political environment

Global health organisations will be more likely to prioritize gender if certain features of their external political environments shift, particularly the acceptance by influential states that improved population health and equity requires addressing gender inequality. This idea is in an early phase of the norm life cycle, having yet to reach a tipping point where a critical mass of states has fully embraced the norm, facilitating a cascade. Momentum is building, however: several of the conditions that facilitate norm emergence are already in place. High-level and effective norm entrepreneurs exist, such as Michelle Bachelet, former Chilean President and present head of the United Nations High Commissioner for Human Rights, and Helen Clark, former Prime Minister of New Zealand and former Administrator of the United Nations Development Programme. Organisational platforms are available, including UN agencies sympathetic to the agenda such as the United Nations Population Fund (UNFPA). Existing norms internalized by many states surrounding human rights present grafting opportunities, or opportunities for leverage. Gender and health is an issue that pertains to bodily integrity, an issue category that historically has resonated internationally. The surge of right-wing populism with a misogynistic bent represents a shock, inspiring social mobilization that may place pressure on states to shift policy. Moreover, there is both historical and recent precedent for the international diffusion of norms relevant to gender equality: the spread of women’s suffrage from the late 1800s through the 1900s, and more recently, the advance of norms on sexual and reproductive health rights and gender equality, facilitated by the 1994 Conference on Population and Development in Cairo and the 1995 Conference on Women in Beijing [80].

### Reforming organisational cultures

The gender and health agenda will also depend on change in organisational cultures. Reform is difficult to enact because of the imprints of founding conditions within global health organizations. Committed top-level leaders are crucial. There is some precedent for leaders playing roles in pushing their organisations to prioritize gender: executive directors Nafis Sadik (1987-2000) and Babatunde Osotimehin (2010-2017) of the UNFPA, and Melinda Gates at the Bill and Melinda Gates Foundation have had such effects. Organisational culture change will also depend on the way professionals are socialized. In-house gender training may play some role, but even more crucial will be shifts in the ways medical schools, economics programs, and public health schools – the institutions that provide many of the professionals that staff these organisations – train their students. There is some evidence that change in training practices is occurring: many such schools are moving away from an exclusive technical and biomedical focus to recognizing the importance of integrating instruction on the structural, social, cultural, and economic forces that shape disease and illness patterns, including gender [81,82].

### Refining proponent strategies

Finally, prospects will be shaped by the decisions gender proponents make regarding defining the issue and pursuing collective action. There are at least three challenges. One concerns problem definition: specifying precisely what the issues are, and how they should be addressed. A strength of the group of gender proponents is diversity. A tension to be managed is how to capitalize on this strength in ways that facilitate cohesion without sidelining voices. Striking a balance is challenging, but it may be facilitated by the global resurgence of right-wing politics, which provides an urgency to finding ways to manage differences and to act with unity.

A second set of challenges pertains to positioning. Among the needs is the articulation of a clear set of demands. The danger in not having such clarity is that leaders of global health organisations will remain confused about what they are being asked to do to address gender. Another positioning challenge concerns the need to address tensions surrounding rights vs instrumentalist framings of the issue. Is it essential to stay true to the principles of human rights in framing the issue? Is the incorporation of economics and other kinds of arguments that may not align with the deeper beliefs of many proponents justifiable as a means of securing allies who may find such rationales more compelling than rights-based arguments? Can a balance be struck between principle-based and instrumental framing strategies? Beyond this, proponents will need to consider how specific problems pertaining to gender, such as violence against women, figure in their framing strategies. Here again is a tension: the agenda is broader than any one issue, yet some issues resonate more widely than others do. Should these issues be used as levers to generate attention, or does doing so marginalize other issues and detract from advancing the broader agenda?

Finally, there is a set of challenges concerning governance. Proponents benefit from their capacity to attract a large, diverse coalition of actors. ICPD provides a historical example of diverse proponents concerned with reproductive health and gender equality effectively building a coalition to place their issues on global and national agendas [83]. However, proponents face difficulties in organizing because they are situated in at least three distinctive although overlapping networks. They will need to attend to the conditions that research indicates facilitate effective network governance – internal legitimacy, external legitimacy, and a balance between stabilization and flexibility. The existing networks’ dispersed nature naturally facilitates flexibility and adaptability – a crucial precondition for network sustainability, especially as it continues to grow and evolve. Proponents will need to figure out how to balance the networks’ malleability with a more formalized and centralized governance arrangement that can facilitate collective action, especially in initial stages where it will be critical for members to manage differences on problem definition and framing strategies.
